# Scalar-on-Function Mode Estimation Using Entropy and Ergodic Properties of Functional Time Series Data

**DOI:** 10.3390/e27060552

**Published:** 2025-05-24

**Authors:** Mohammed B. Alamari, Fatimah A. Almulhim, Ibrahim M. Almanjahie, Salim Bouzebda, Ali Laksaci

**Affiliations:** 1 Department of Mathematics, College of Science, King Khalid University, Abha 62223, Saudi Arabia; 2 Department of Mathematical Sciences, College of Science, Princess Nourah bint Abdulrahman University, P.O. Box 84428, Riyadh 11671, Saudi Arabia; 3 Université de Technologie de Compiègne, LMAC (Laboratory of Applied Mathematics of Compiègne), 60203 Compiègne, France

**Keywords:** $L^{1}$-modal regression, functional data, ergodic data, recursive estimate, nonparametric prediction, complete consistency, conditional mode, quantile regression, 62G08, 62G10, 62G35, 62G07, 62G32, 62G30, 62H12

## Abstract

In this paper, we investigate the recursive $L^{1}$ estimator of the conditional mode when the input variable takes values in a pseudo-metric space. The new proposed estimator is constructed under an ergodicity assumption, which provides a robust alternative to the standard mixing processes in various practical settings. The particular interest of this contribution arises from the difficulty in incorporating the mathematical properties of a functional mixing process. In contrast, ergodicity is characterized by the Kolmogorov–Sinai entropy, which measures the dynamics, the sparsity, and the microscopic fluctuations of the functional process. Using an observation sampled from ergodic functional time series (fts), we establish the asymptotic properties of this estimator. In particular, we derive its convergence rate and show Borel–Cantelli (BC) consistency. The general expression for the convergence rate is then specialized to several notable scenarios, including the independence case, the classical kernel method, and the vector-valued case. Finally, numerical experiments on both simulated and real-world datasets demonstrate the superiority of the $L^{1}$-recursive estimator compared to existing competitors.

## 1. Introduction

Investigating the joint behavior of two random variables in a functional setting is an active area of applied statistics, as it facilitates quantifying the influence of a functional covariate on a scalar response. Numerous functional approaches have been proposed to capture this relationship, including conditional expectation, relative regression, and median regression. However, modeling the relationship via the conditional distribution function is often regarded as more informative because it sheds light on both central and extreme parts of the response. Consequently, the principal goal of this work is to introduce a new estimator for modal regression using the cumulative distribution function.

Despite substantial literature on conditional mode prediction, the predominant estimator remains the Nadaraya–Watson (NW) method. The earliest investigation of conditional mode estimation can be traced back to [1], which demonstrated that the mode can yield superior predictive performance compared with the conditional mean. In [2], the authors proposed a mode-based predictor using the derivative of the conditional density, applicable to vector-valued input variables. Subsequently, ref. [3] derived the asymptotic distribution of the modal regression estimator under independence, and this result was generalized to dependent data by [4]. For more recent works, we refer the readers to [5].

A functional version of the NW-estimator for the conditional mode (CM) was first introduced in [6], where the authors established almost complete consistency of the estimator by identifying it as the maximizer of the conditional density. This result was extended to dependent processes in [7]. The asymptotic distribution of the NW-based functional CM estimator was studied under the i.i.d. assumption in [8], whereas [9] considered strong mixing functional time series under fractal conditions. The monograph of [10] represents a key contribution to nonparametric functional prediction, and further theoretical results on functional mode estimation can be found in [11], which addressed the $L^{p}$-convergence of NW-based functional mode estimators. In the context of ergodic functional time series, ref. [12] focused on conditional mode estimation and derived BC consistency under a missing-at-random framework for the functional covariate. Several alternative estimators to the NW approach have also emerged in functional data analysis (FDA). For example, ref. [13] established the asymptotic normality of a local linear CM estimator in the functional setting, ref. [14] investigated a kNN-based functional CM approach, and [15] developed a local linear functional-kNN version of the estimator. More extensive studies on functional CM estimation can be found in [16,17,18] and related references. Additionally, in the ergodic functional time series case, ref. [19] obtained BC consistency for the conditional mode estimator.

A distinctive contribution of the present paper is its focus on a recursive estimation algorithm, a direction that remains underexplored in FDA. One of the earliest examinations of recursive methods in this domain is [20], which addressed the recursive estimation of conditional mean functions. Later, ref. [21] investigated recursive procedures for functional time series under mixing conditions. More recent developments in functional nonparametric smoothing by means of recursive algorithms, along with relevant references, are presented in [22]. For additional perspectives on FDA and its applications, including dedicated survey articles and specialized journal issues, see [23,24,25,26], among others and recent papers [27,28,29].

### 1.1. Contributions of This Paper

The primary objective of this work is to propose a novel modal regression estimator and establish its asymptotic properties under a general framework of ergodic functional time series. Specifically, our estimator combines an $L^{1}$-based approach with a recursive procedure. In contrast to estimators built upon the NW or local linear methods, the newly constructed estimator offers multiple advantages. First, incorporating an $L^{1}$-technique promotes robustness, which mitigates the impact of outliers through a percentile-based approach. Moreover, harnessing the conditional distribution function to identify the conditional mode leverages comprehensive information about the functional covariate–response relationship, potentially enhancing the estimator’s precision. A further strength lies in the recursive structure, which seamlessly updates the estimator upon the arrival of each new data point—a feature especially valuable for real-time forecasting in ergodic functional time series. This adaptability is highly relevant in fields such as artificial intelligence, where continuous data processing is critical. From a theoretical standpoint, we derive the Borel–Cantelli convergence rate of the proposed estimator under mild ergodic conditions frequently satisfied by common processes (e.g., moving average (MA), generalized autoregressive conditional heteroskedasticity (GARCH), Volterra). Finally, we illustrate the practical value of our algorithm through empirical investigations on both synthetic and real-world datasets.

### 1.2. Paper Organization

We introduce the $L^{1}$-based conditional mode and its recursive estimator in Section 2. The main theoretical results, including consistency and convergence rates, are presented in Section 3. Section 4 is devoted to some discussion on the real impact of the principal axes of the studied topic. In Section 5, we investigate the finite-sample performance of the proposed estimator through simulation studies and applications to real data. The proofs of the auxiliary results are provided in Section 6.

## 2. The $L^{1}$-Recursive Estimation of the Mode

Consider a strictly stationary sequence of dependent input–output random variables, denoted by ${\left(I_{i},O_{i}\right)}_{i=1,\ldots ,n}$, which takes values in F×R. Here, F is a semi-metric space endowed with a semi-metric d(·,·). Let $N_{\theta }$ be a neighborhood of a fixed curve θ∈F. We assume that the conditional distribution function G(·∣θ) is strictly increasing and admits a continuous density g(y∣θ) with respect to the Lebesgue measure on R. Recall that for a given p∈(0,1), the conditional quantile of *O* given I=θ, denoted by $Qu_{p}\left(\theta \right)$, is obtained by inverting the conditional distribution function, namely

$$Qu_{p}\left(\theta \right)\;=\;G^{-1}\left(p\;|\;\theta \right).$$

Meanwhile, the conditional mode of *O* given I=θ, denoted by CM(θ), is defined as the maximizer of the conditional density on a given compact set K⊂R:

$$CM\left(\theta \right)\;=\;\arg\max_{y\in K}\;g\left(y|\theta \right).$$

By combining these two notions, one can re-express modal regression as

(1) $$CM\left(\theta \right)\;=\;Qu_{p_{\theta }}\left(\theta \right)\;\mathrm{with}\;p_{\theta }\;=\;\arg\min_{\;p\;\in \;G^{-1}\left(K|\theta \right)}\frac{\partial }{\partial p}\;Qu_{p}\left(\theta \right).$$

In the rest of this paper, we assume that the compact subset *K* locates one conditional mode CM(θ) and that (1) holds.

The $L^{1}$-estimator of the conditional mode naturally connects to the $L^{1}$-quantile regression, which is determined by

$$Qu_{p}\left(\theta \right)\;=\;\arg\min_{t\in R}\Psi_{p}\left(\theta ,t\right),$$

where

$$\Psi_{p}\left(\theta ,t\right)\;=\;E\left[L_{p}\left(O-t\right)\;|\;I=\theta \right]\;\mathrm{and}\;L_{p}\left(s\right)\;=\;\left(2p-1\right)\;s\;+\;\left|s\right|.$$

An $L^{1}$-recursive estimator of the function $Qu_{p}\left(\cdot \right)$ can thus be defined by

(2) $$\hat{Qu_{p}}\left(\theta \right)\;=\;\arg\min_{t\in R}\hat{\Psi_{p}}\left(\theta ,t\right),$$

where

$$\hat{\Psi_{p}}\left(\theta ,t\right)\;=\;\frac{\sum_{i=1}^{n}\Gamma \left(a_{i}^{-1}\;d\left(\theta ,I_{i}\right)\right)\;\left[\;\left(2p-1\right)\;\left(O_{i}-t\right)\;+\;\left|\;O_{i}-t\;\right|\right]}{\sum_{i=1}^{n}\Gamma \left(a_{i}^{-1}\;d\left(\theta ,I_{i}\right)\right)},\;\forall \;t\in R,$$

where Γ is a kernel function, and $\left\{a_{i}\right\}$ is a sequence of positive real numbers satisfying $\lim_{n\to \infty }a_{n}=0$. Note that in the recursive estimation setting, in contrast to the Nadaraya–Watson-type (NW) approach, the bandwidth $a_{i}$ depends on the specific input observations $\left\{I_{i}\right\}$, thereby allowing the estimator to be updated whenever a new observation is obtained.

Before constructing a $L^{1}$-recursive estimator of the modal regression, it is necessary to define estimators for both $p_{\theta }$ and $Qu_{p}^{\prime }\left(\theta \right)$. Recall that

$$Qu_{p}^{\prime }\left(\theta \right)\;=\;\frac{\partial }{\partial p}\;Qu_{p}\left(\theta \right)\;=\;\lim_{\;b\to 0}\;\frac{Qu_{\;p+b}\left(\theta \right)\;-\;Qu_{p}\left(\theta \right)}{b}.$$

A natural estimator for $Qu_{p}^{\prime }\left(\theta \right)$ is then given by

$$\hat{Qu_{p}^{\prime }}\left(\theta \right)\;=\;\frac{\hat{Qu_{p+h_{n}}}\left(\theta \right)\;-\;\hat{Qu_{p-h_{n}}}\left(\theta \right)}{2\;h_{n}},$$

where $\left\{h_{n}\right\}$ is a sequence of positive real numbers converging to 0. The conditional mode CM(θ) is accordingly estimated by

(3) $$\hat{CM}\left(\theta \right)\;=\;\hat{Qu_{\hat{p_{\theta }}}}\left(\theta \right),$$

where

$$\hat{p_{\theta }}\;=\;\arg\min_{\;p\;\in \;G^{-1}\left(K|\theta \right)}\hat{Qu_{p}^{\prime }}\left(\theta \right).$$

Of course, $\hat{CM}\left(\theta \right)$ is not necessarily unique, and if that is the case, $\hat{CM}\left(\theta \right)$ will concern any values verifying (3).

In the theoretical development, our principal goal is to establish a Borel–Cantelli convergence result for $\hat{CM}\left(\theta \right)$. To achieve this, we adopt the classical ergodicity assumption, which is more general than ordinary mixing conditions. A process is typically considered ergodic if, over sufficient time, the entropy measured along a single evolving trajectory converges to the entropy of the system’s full ensemble of possible states. In the functional case, we employ the definition of ergodicity for functional statistics proposed by [7], as it provides a suitable framework for analyzing dependent functional data.

## 3. Main Results

We begin by letting *C* or $C^{\prime }$ denote generic strictly positive constants. We also assume that

$$G^{-1}\left(K|\theta \right)\;=\;\left[a_{\theta },\;b_{\theta }\right].$$

Moreover, for each k=1,…,n, define $G_{k}$ as the σ-field generated by $(\left(I_{1},O_{1}\right),\ldots$, $\left(I_{k},O_{k}\right),I_{k+1})$, and let $F_{k}$ be the σ-field generated by $\left(\left(I_{1},O_{1}\right),\ldots ,\left(I_{k},O_{k}\right)\right)$.

Our principal assumptions are as follows:

(**Co**1) $$\left\{\begin{array}{c} \left(i\right)\;\mathrm{The}\;\mathrm{function}\;\xi \left(\theta ,a\right)\;:=\;I\;P\left(I\in B(\theta ,a)\right)\;\mathrm{satisfies}\;\xi \left(\theta ,a\right)>0\;\mathrm{for}\;\mathrm{every}\;a>0,\\ \;\mathrm{where}\;B\left(\theta ,r\right)=\{\theta^{\prime }\in F:d\left(\theta^{\prime },\theta \right)<r\}.\\ \left(\mathrm{ii}\right)\;\mathrm{For}\;\mathrm{each}\;i=1,\ldots ,n,\;\mathrm{there}\;\mathrm{exists}\;a\;\mathrm{deterministic}\;\mathrm{function}\;\xi_{i}\left(\theta ,\cdot \right)\;\mathrm{such}\;\mathrm{that}\\ \;\mathrm{almost}\;\mathrm{surely},\;0<I\;P\left(I_{i}\in B\left(\theta ,a\right)|F_{i-1}\right)\;\leq \;\xi_{i}\left(\theta ,a\right),\;\forall \;a>0,\\ \;\mathrm{and}\;\xi_{i}\left(\theta ,a\right)\to 0\;\mathrm{as}\;a\to 0.\\ \left(\mathrm{iii}\right)\;\mathrm{For}\;\mathrm{any}\;\mathrm{positive}\;\mathrm{sequence}\;{\left(a_{i}\right)}_{i=1,\ldots ,n},\;\mathrm{we}\;\mathrm{have}\\ \;\frac{\sum_{i=1}^{n}I\;P\left(I_{i}\in B\left(\theta ,a_{i}\right)|F_{i-1}\right)}{\sum_{i=1}^{n}\xi \left(\theta ,a_{i}\right)}\;⟶1\;a.\mathrm{co}. \end{array}\right.$$
(**Co**2) The function $Qu_{\cdot }\left(\theta \right)$ is three times continuously differentiable on $\left[a_{\theta },b_{\theta }\right]$. In addition, suppose that G(·∣·) satisfies the Lipschitz condition
  $$\;\forall \;\theta_{1},\theta_{2}\in N_{\theta },\;\;\forall \;t_{1},t_{2}\in \left[a_{\theta },b_{\theta }\right],\;\left|G\left(t_{1}|\theta_{1}\right)-G\left(t_{2}|\theta_{2}\right)\right|\;\leq \;C\;d^{b}\left(\theta_{1},\theta_{2}\right)\;+\;\left|t_{1}-t_{2}\right|,$$
  for some b>0, where $N_{\theta }$ is a neighborhood of θ.
(**Co**3) The function Γ is supported on (0,1) and fulfills
  $$0\;<\;C\;{1\;I}_{\left(0,1\right)}<\Gamma (t)<C^{\prime }\;{1\;I}_{\left(0,1\right)}<\infty .$$
(**Co**4) $$\lim_{n\to \infty }\;\frac{\zeta_{n}\;\ln n}{\;n^{2}\;\xi_{n}^{2}\;}\;=\;0,\;\mathrm{where}\;\zeta_{n}\;=\;\sum_{i=1}^{n}\xi_{i}\left(\theta ,a_{i}\right)\;\mathrm{and}\;\xi_{n}\;=\;\frac{1}{n}\sum_{i=1}^{n}\xi \left(\theta ,a_{i}\right).$$

Clearly, conditions (**Co**1)–(**Co**4) are often encountered in nonparametric functional time series analysis. In particular, (**Co**1) describes the probabilistic concentration behavior of the input variable, including its conditional concentration with respect to the filtration, which underscores the impact of ergodicity on the asymptotic properties of the estimator. Assumption (**Co**2) is pivotal for the nonparametric structure of the model. Conditions (**Co**3) and (**Co**4) govern the behavior of the kernel Γ and the smoothing parameters $a_{n}$ and $h_{n}$, ensuring the proper handling of the technical aspects of the estimator $\hat{CM}\left(\theta \right)$. These requirements also allow us to express the convergence rate in a form analogous to the Nadaraya–Watson estimator, which can be viewed as arising from maximizing a double-kernel estimator of a conditional density. Thus, the assumptions under consideration effectively encompass the main components of the subject—namely, the model, the data structure, the correlation framework, and the convergence rate. These assumptions are not overly restrictive, especially given the complexity of the proposed functional time series model and the strength of the Borel–Cantelli (BC) type consistency. In fact, it is possible to establish a weaker form of consistency for the estimator under less stringent conditions. By employing techniques similar to those presented in [30], one can demonstrate weak consistency. Ultimately, the relationship between the assumptions and the resulting theoretical guarantees reflects a trade-off: stronger and more general results necessitate stronger assumptions. The next theorem establishes the almost-complete (a.co.) convergence of $\hat{CM}\left(\theta \right)$.

**Theorem 1.**  *Suppose* (**Co**1)–(**Co**4) *hold. If*

$$\underset{\;p\;\in \;(0,1)}{\inf}\;\frac{\partial^{3}}{\partial p^{3}}\;Qu_{p}\left(\theta \right)\;>\;0,$$

*then*

$$\hat{CM}\left(\theta \right)\;-\;CM\left(\theta \right)\;=\;O\;\left(\frac{1}{\zeta_{n}}\;\sum_{i=1}^{n}a_{i}^{\;b}\;\xi_{i}\left(\theta ,a_{i}\right)\right)\;+\;O\;\left(h_{n}\right)\;+\;O\;\left(\sqrt{\frac{\zeta_{n}\;\ln n}{\;n^{2}\;\xi_{n}^{2}\;}}\right),\;a.\mathrm{co}.$$

**Proof of the Main Result.**  From the definitions of $\hat{CM}\left(\theta \right)$ and CM(θ), it follows that

(4) $$\begin{array}{ccc} \left|\hat{CM}\left(\theta \right)\;-\;CM\left(\theta \right)\right|\; & = & \;\left|\hat{Qu_{\hat{p_{\theta }}}}\left(\theta \right)\;-\;Qu_{p_{\theta }}\left(\theta \right)\right|\\ \; & = & \;\left|\hat{Qu_{\hat{p_{\theta }}}}\left(\theta \right)-Qu_{\hat{p_{\theta }}}\left(\theta \right)\;+\;Qu_{\hat{p_{\theta }}}\left(\theta \right)-Qu_{p_{\theta }}\left(\theta \right)\right|\\ \; & \leq & \;\underset{\;p\;\in \;[a_{\theta },b_{\theta }]}{\sup}\left|{\hat{Qu}}_{p}\left(\theta \right)-Qu_{p}\left(\theta \right)\right| \end{array}$$

(5) $$\begin{array}{ccc} \; & \; & \;+\;\underset{\;p\;\in \;[a_{\theta },b_{\theta }]}{\sup}\left|\;Qu_{\hat{p_{\theta }}}\left(\theta \right)-Qu_{p_{\theta }}\left(\theta \right)\right|. \end{array}$$

Using a Taylor expansion, we have

$$Qu_{\hat{p_{\theta }}}\left(\theta \right)\;-\;Qu_{p_{\theta }}\left(\theta \right)\;=\;\left(\hat{p_{\theta }}-p_{\theta }\right)\;{\left\{Qu^{\prime }\right\}}_{p_{\theta }^{*}}\left(\theta \right),\;\mathrm{for}\;\mathrm{some}\;\;p_{\theta }^{*}\in \left(\hat{p_{\theta }},\;p_{\theta }\right).$$

Since $p_{\theta }$ is the minimizer of $Qu^{\prime }\left(\cdot |\theta \right)$, we also obtain

$$Qu_{\hat{p_{\theta }}}^{\prime }\left(\theta \right)\;-\;Qu_{p_{\theta }}^{\prime }\left(\theta \right)\;=\;{\left(\hat{p_{\theta }}-p_{\theta }\right)}^{2}\;{\left\{Qu^{\prime \prime \prime }\right\}}_{p_{\theta }^{**}}\left(\theta \right),\;\mathrm{for}\;\mathrm{some}\;\;p_{\theta }^{**}\in \left(\hat{p_{\theta }},\;p_{\theta }\right).$$

Analogously to (4), it follows that

$$\left|\;Qu_{\hat{p_{\theta }}}^{\prime }\left(\theta \right)-Qu_{p_{\theta }}^{\prime }\left(\theta \right)\right|\;\leq \;2\;\underset{\;p\;\in \;[a_{\theta },b_{\theta }]}{\sup}\left|{\hat{Qu^{\prime }}}_{p}\left(\theta \right)-Qu_{p}^{\prime }\left(\theta \right)\right|.$$

Because $\underset{p\in (0,1)}{\inf}\frac{\partial^{3}}{\partial p^{3}}\;Qu_{p}\left(\theta \right)\;>\;0,$ we obtain

(6) $$\hat{p_{\theta }}-p_{\theta }\;\leq \;C\;\sqrt{\;\underset{p\;\in \;[a_{\theta },b_{\theta }]}{\sup}\left|{\hat{Qu^{\prime }}}_{p}\left(\theta \right)-Qu_{p}^{\prime }\left(\theta \right)\right|}.$$

Combine (4) and (6) to obtain

(7) $$\begin{array}{c} \left|\hat{CM}\left(\theta \right)-CM\left(\theta \right)\right|\\ \;\leq \;C\;\left(\underset{p\;\in \;[a_{\theta },b_{\theta }]}{\sup}\left|{\hat{Qu}}_{p}\left(\theta \right)-Qu_{p}\left(\theta \right)\right|\;+\;\sqrt{\underset{p\;\in \;[a_{\theta },b_{\theta }]}{\sup}\left|{\hat{Qu^{\prime }}}_{p}\left(\theta \right)-Qu_{p}^{\prime }\left(\theta \right)\right|}\right). \end{array}$$

Hence, determining the convergence rate reduces to studying

$$\underset{p\;\in \;[a_{\theta },b_{\theta }]}{\sup}\left|{\hat{Qu}}_{p}\left(\theta \right)-Qu_{p}\left(\theta \right)\right|\;\mathrm{and}\;\underset{p\;\in \;[a_{\theta },b_{\theta }]}{\sup}\left|{\hat{Qu^{\prime }}}_{p}\left(\theta \right)-Qu_{p}^{\prime }\left(\theta \right)\right|.$$

Furthermore, asymptotically,

$$\begin{array}{c} \hat{Qu_{p}^{\prime }}\left(\theta \right)-Qu_{p}^{\prime }\left(\theta \right)\\ \;=\;\frac{\;\hat{Qu_{p+h_{n}}}\left(\theta \right)-Qu_{p+h_{n}}\left(\theta \right)\;+\;Qu_{p-h_{n}}\left(\theta \right)-\hat{Qu_{p-h_{n}}}\left(\theta \right)\;}{2h_{n}}\\ \;+\;\frac{\;Qu_{p+h_{n}}\left(\theta \right)-Qu_{p}\left(\theta \right)\;+\;Qu_{p}\left(\theta \right)-Qu_{p-h_{n}}\left(\theta \right)\;-\;2h_{n}\;Qu_{p}^{\prime }\left(\theta \right)\;}{2h_{n}}\\ \;\leq \;C\;h_{n}^{-1}\;\underset{q\;\in \;(a_{\theta }-h_{n},\;b_{\theta }+h_{n}]}{\sup}\left|\hat{Qu_{q}}\left(\theta \right)-Qu_{q}\left(\theta \right)\right|\;+\;O\left(h_{n}\right). \end{array}$$

Finally, Theorem 1 follows from the following lemmas. □

**Lemma** **1** **([**16**])**. *Consider a family of real-valued random functions $\left\{B_{n}\right\}$, each of which is decreasing in γ. Let $\left\{A_{n}\right\}$ be a real-valued random sequence. Suppose there exist positive constants λ,M>0 such that*

$$A_{n}=o_{a.co.}\left(1\right)\;\mathrm{and}\;\underset{|\gamma |\leq M}{\sup}\left|B_{n}\left(\gamma \right)+\lambda \gamma -A_{n}\right|=o_{a.co.}\left(1\right).$$

*Then, for any real random sequence $\left\{\gamma_{n}\right\}$ satisfying $B_{n}\left(\gamma_{n}\right)=o_{a.co.}\left(1\right)$, it follows that*

$$\sum_{n=1}^{\infty }P\left\{|\gamma_{n}|\geq M\right\}<\infty .$$

**Lemma 2.**  *Suppose* (**Co1**) *and* (**Co3**)–(**Co4**) *hold. Then, we have*

$${\hat{Q}}_{D}\left(\theta \right)-{\bar{Q}}_{D}\left(\theta \right)=O\left(\sqrt{\frac{\zeta_{n}\;\ln n}{n^{2}\;\xi_{n}^{2}}}\right),\;a.co.$$

*Moreover, there exists a constant C>0 such that*

$$\sum_{n}I\;P\left({\bar{Q}}_{D}\left(\theta \right)<C\right)<\infty .$$

*Here,*

$${\hat{Q}}_{D}\left(\theta \right):=\frac{1}{n\;\xi_{n}}\sum_{i=1}^{n}\Gamma \left(a_{i}^{-1}\;d\left(\theta ,I_{i}\right)\right)$$

*and*

$${\bar{Q}}_{D}\left(\theta \right):=\frac{1}{n\;\xi_{n}}\sum_{i=1}^{n}I\;E\left[\Gamma \left(a_{i}^{-1}\;d\left(\theta ,I_{i}\right)\right)\;|\;F_{i-1}\right].$$

**Proposition 1.**  *Assume* (**Co1**)–(**Co4**) *hold. Then, there is a positive constant λ such that*

$${\hat{Qu}}_{p}\left(\theta \right)-Qu_{p}\left(\theta \right)=\frac{1}{g\left(Qu_{p}|\theta \right)}\;A_{n}+O\left(\underset{|\gamma |\leq M}{\sup}\left|B_{n}\left(\gamma \right)+\lambda \;\gamma -A_{n}\right|\right),$$

*where*

$$B_{n}\left(\gamma \right)=\frac{1}{n\;\xi_{n}}\sum_{i=1}^{n}\left[p-1_{\left\{O_{i}\leq \gamma +Qu_{p}\left(\theta \right)\right\}}\right]\Gamma_{i},\;\mathrm{and}\;A_{n}=B_{n}\left(0\right).$$

**Proposition 2.**  *Under the same assumptions* (**Co1**)–(**Co4**)*, we also have*

$$\underset{p\in (0,1)}{\sup}\left|{\hat{Qu}}_{p}\left(\theta \right)-Qu_{p}\left(\theta \right)\right|=O\left(\frac{1}{\zeta_{n}}\sum_{i=1}^{n}a_{i}^{\;b}\;\xi_{i}\left(\theta ,a_{i}\right)\right)+O\left(\sqrt{\frac{\zeta_{n}\;\ln n}{n^{2}\;\xi_{n}^{2}}}\right),\;a.co.$$

## 4. Discussion and Comments

### 4.1. On the Ergodic Functional Time Series

Similarly to multivariate statistics, ergodicity plays a crucial role in functional statistics. In particular, ergodicity ensures that temporal averages converge to their corresponding stochastic means. This property is especially important, as it justifies the use of sample mean and covariance functions as consistent estimators of the true mean function and the true covariance operator. These estimators, in turn, allow for efficient estimation of eigenfunctions in functional principal component analysis (FPCA) and for accurate curve smoothing using a chosen basis, such as splines or Fourier functions. All these methodologies fundamentally rely on the sample mean and the empirical covariance operator. The ergodic behavior and functional characteristics of the time series under consideration are governed by assumption (**Co1**), which quantifies the concentration properties of the functional variables. This assumption is thoroughly discussed in [6], where it is shown that **Co1(i)** holds for a wide class of continuous processes whose probability measures are absolutely continuous with respect to the Wiener measure. Examples include the Poisson process, the Ornstein–Uhlenbeck process, fractional Brownian motion, and general diffusion processes. In this work, we also focus on the conditional version of this assumption, namely **(Co1)(ii–iii)**. This extension enables us to account for the dependence structure of the process by analyzing its long-memory behavior, a standard approach in dynamic systems modeling and time series analysis. In such contexts, conditional distributions with respect to the past filtration $F_{i-1}$ are frequently employed to control process evolution, verify the martingale property, and assess predictability. Using arguments similar to those used in the unconditional case **(Co1)(i)**, one can show that a trivial example of a functional ergodic process satisfying **(Co1)(ii–iii)** is when its conditional distribution, given the past, is absolutely continuous with respect to the Wiener measure. Additionally, the Karhunen–Loève decomposition can be used to represent such processes explicitly (see [6] for examples of functional processes admitting such a decomposition). It is worth emphasizing that while both mixing and ergodicity describe forms of dependence among observations, they are fundamentally different. Specifically, the mixing property implies that any two subsets of the state space become asymptotically independent over time, whereas ergodicity implies that the system’s trajectory visits all regions of the space in proportion to their probability measure. Importantly, ergodicity is generally easier to verify than mixing. It is well known that ergodicity does not imply mixing, and there exist numerous ergodic time series that fail to satisfy any form of mixing assumption. Prominent examples include the following:

– First-order autoregressive processes with Bernoulli innovations (see [31]);
– Gaussian processes with Hurst exponent H>0.5 (see [32]);
– Gaussian processes with non-decaying covariance structures (see [33]).

Additional examples are discussed in [34], and these models can be naturally extended to the functional setting.

### 4.2. The Conditional Mode Versus the Conditional Mean

In predictive modeling, particularly when the conditional distribution is asymmetric or multimodal, the conditional mode often yields more accurate and meaningful predictions than the conditional mean. While the conditional mean represents the average outcome given certain inputs, it can be significantly affected by outliers or skewness in the distribution. In contrast, the conditional mode reflects the most probable outcome, making it more robust, reliable, and informative. A similar conclusion applies to the conditional median, which is also less sensitive to extreme values than the mean. As a result, combining the conditional mode and median can significantly outperform the conditional mean in predictive tasks. This advantage becomes even more crucial in the context of ergodic functional time series, where providing robust predictors is essential. Ergodicity ensures that time averages converge to ensemble averages, offering a sound statistical basis for long-term forecasting. Moreover, the environmental data under study often exhibits seasonality, which can distort conditional mean predictions. In particular, repeating seasonal patterns can oversmooth the conditional expectation, reducing forecasting accuracy. The conditional mode, however, better captures the most likely outcomes within each seasonal segment, making it especially suitable for forecasting applications.

### 4.3. The Recursive Estimation in Action

As with all smoothing approaches, the choice of the bandwidth parameter $a_{i}$ is critical to the quality of the estimation. Typically, the mean squared error serves as a fundamental criterion for selecting this parameter. In the recursive framework considered here, we adopt the selection algorithm proposed by [20]. The smoothing parameter $a_{i}$ is fixed to $a_{i}=Ci^{-\upsilon }$, where

$$C=\max d_{i}\left(\theta ,I_{i}\right),$$

and υ is selected by the cross-validation rule as follows

(8) $$\upsilon_{opt}=\arg\min_{\upsilon \in (0,1)}\sum_{j=1}^{n}{\left(O_{j}-{\hat{CM}}^{-j}\left(I_{j}\right)\right)}^{2},$$

where ${\hat{CM}}^{-j}$ is the leave-out-one estimator of the estimator. The rule (8) is similar to the cross-validation criterion considered by [10]. In our empirical analysis, the rule (8) is optimized over *m* equidistant points in the interval (0,1). Finally, it is worth noting that, although this selection approach has demonstrated good empirical performance, establishing its asymptotic optimality remains an important direction for future research.

### 4.4. The Computational Cost

Recall that, unlike traditional kernel estimators, which compute the estimate independently at each point, the recursive version updates the estimate sequentially with each new observation, potentially reducing execution time. Consequently, computational efficiency is a significant advantage of the recursive estimator. Quantifying this efficiency is particularly important in the context of large datasets or real-time applications. Specifically, if each update involves a constant number of operations, i.e., of order O(1), then the total computational cost becomes O(n), which is considerably more efficient than the $O(n^{2})$ complexity of standard kernel estimators. However, in practical scenarios where the bandwidth is selected via adaptive tuning, the computational cost may increase. Despite its advantages, the recursive approach has a notable drawback: it requires storing past data, which negatively impacts memory usage. This limitation becomes especially critical when dealing with large sample sizes or high-dimensional data.

## 5. Simulation Study

In this simulation study, our objective is to investigate the feasibility and effectiveness of the proposed method. In particular, we seek to assess how dependence impacts the convergence rate comparing the algorithm’s performance under different scenarios such as varying dependence levels, signal-to-noise ratios, or outlier contamination. To achieve this, we first generate an artificial dataset following a nonparametric form:

$$\begin{array}{ccc} \mathrm{heteroscedastic}\;\left(\mathrm{Het}.\right)\;\mathrm{Model}:O_{i} & = & 4\int_{0}^{1}\sin\left(3+I_{i}^{3}\left(t\right)\right)\;dt\;+\;\cos\left(3+I_{i}^{3}\left(t\right)\right)\;\epsilon_{i},\\ \mathrm{homoscedastic}\;\left(\mathrm{Hom}.\right)\;\mathrm{Model}:O_{i} & = & 5\int_{0}^{1}\log\left(\frac{2+I_{i}^{2}\left(t\right)}{3+I_{i}^{3}\left(t\right)}\right)\;dt\;+\;\epsilon_{i}, \end{array}$$

and heteroscedastic model with signal-to-noise ratio (SNRHet.) Model:

$$O_{i}=4\int_{0}^{1}\sin\left(3+I_{i}^{3}\left(t\right)\right)\;dt\;+\;\sigma_{SNR}\;\epsilon_{i},$$

where $\epsilon_{i}$ and $I_{i}$ are independent, and $\sigma_{SNR}$ is controlled by considering various values of signal-to-noise ratio $SNR_{k}=5\%$ and 40%, where

$$SNR_{k}=\frac{\sigma_{SNR}^{2}}{\frac{1}{n}\sum_{i=1}^{n}{\left(R_{i}-\bar{R}\right)}^{2}},\;R_{i}=4\int_{0}^{1}\sin\left(3+I_{i}^{3}\left(t\right)\right).$$

**Figure 1:**
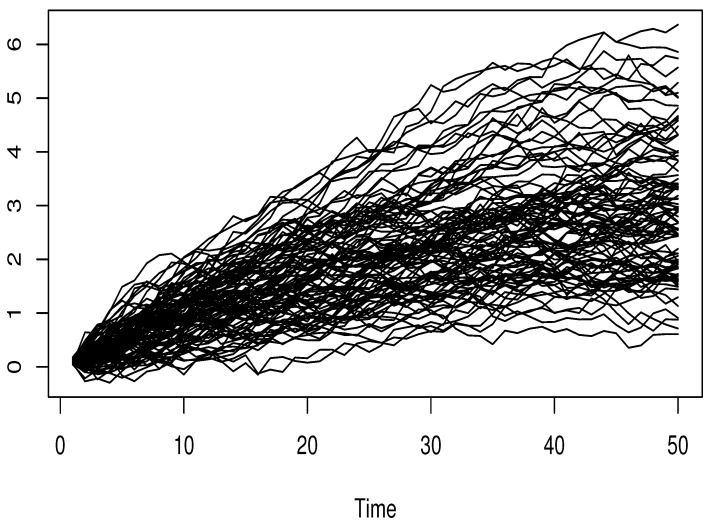
Functional autoregressive order 2: Wiener kernel.

**Figure 2:**
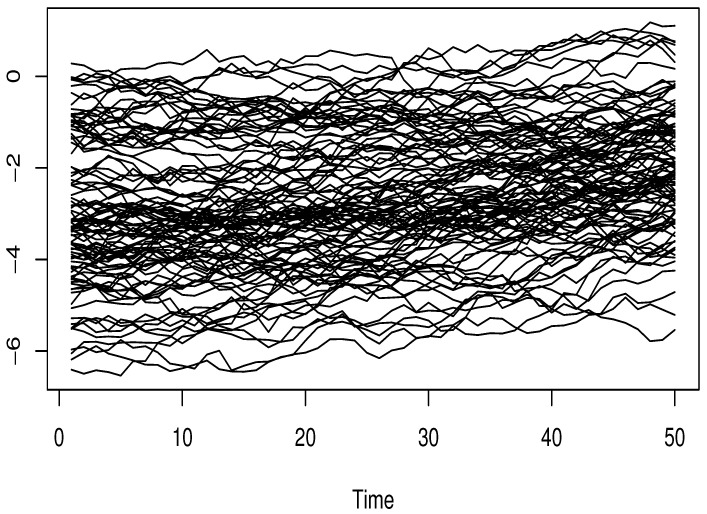
Functional autoregressive order 2: Gaussian kernel.

**Figure 3:**
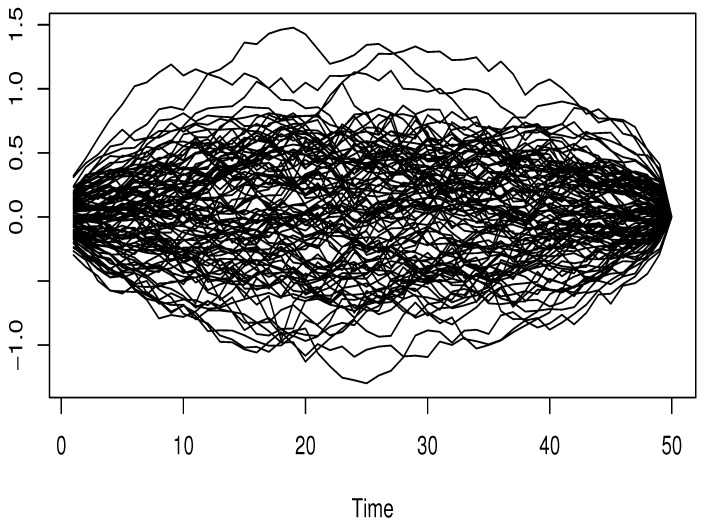
Functional GRCH order 1.

The functional input variable is generated from dependent functional processes using the R-package *FTSgof* (https://www.r-project.org/). We have generated n=150 observations. The resulting functional variables are presented in Figure 1, Figure 2 and Figure 3.

Clearly, this sampling process encompasses three types of FTS-dependence, specifically functional autoregressive processes of order 2 (FAR(2)) involving two distinct kernels

$$\mathrm{Gaussian}\;\mathrm{kernel}\;\psi \left(t,s\right)=\exp\left\{\frac{t^{2}+s^{2}}{2}\right\}\;\;t,s\in \left[0,1\right],$$

Wienerkernelψ(t,s)=t(1−t)s(1−s)t,s∈[0,1].

The third illustration of a functional covariate setting is the functional ARCH(1) model, whose conditional volatility depends on the following kernel:

(9) Defaultkernelα(t,s)=12t(1−t)s(1−s),t,s∈[0,1].

This kernel is the default choice in the *FTSgof R*-package. In our experimental design, the correlation level is adjusted via the function *fACF*. It is evident that this aspect emphasizes the effect of dependency levels on the accuracy of estimates. Meanwhile, the impact of outliers is managed by adjusting the observation responses ${\left(O_{i}\right)}_{i}$ with a multiplicative factor MF, which can be either MF=1 or MF=10. Meanwhile, the true values of the conditional mode, denoted by CM(θ), are obtained from the distribution of the underlying white noise $\epsilon_{i}$. This step is crucial because it allows us to evaluate how the nonparametric component influences the prediction task.

To investigate robustness, we consider three distinct distributions: Weibull, Laplace, and Log normal. These distributions are selected for their invariance under translation and varying degrees of heavy-tailed behavior, which in turn help to gauge the estimator’s resilience to outliers. We then compare the $L^{1}$-robustness of our estimator against multiple existing predictors.

To examine how recursion affects estimation, we contrast our proposed recursive estimator with a non-recursive version, in which the bandwidth parameter $a_{i}$ remains fixed at $a_{n}$. We further assess robustness by comparing $\hat{CM}$ against the double-kernel (DK) estimators given by

(10) $$\mathrm{The}\;\mathrm{NW}\text{-}\mathrm{estimator}:\;\tilde{CM}\left(\theta \right)=\arg\max_{y}\frac{\sum_{i=1}^{n}\Gamma \;\left(a_{n}^{-1}d\left(\theta ,I_{i}\right)\right)\;\Gamma \;\left(b_{n}^{-1}\left(y-O_{i}\right)\right)}{\sum_{i=1}^{n}b_{n}\;\Gamma \;\left(a_{n}^{-1}d\left(\theta ,I_{i}\right)\right)},$$

and

(11) $$\mathrm{The}\;\mathrm{DK}\text{-}\mathrm{recursive}\;\mathrm{estimator}:\bar{CM}\left(\theta \right)=\arg\max_{y}\frac{\sum_{i=1}^{n}\Gamma \;\left(a_{i}^{-1}d\left(\theta ,I_{i}\right)\right)\;\Gamma \;\left(b_{n}^{-1}\left(y-O_{i}\right)\right)}{\sum_{i=1}^{n}b_{n}\;\Gamma \;\left(a_{i}^{-1}d\left(\theta ,I_{i}\right)\right)}.$$

The performance of these three estimators, $\hat{CM}$, $\tilde{CM}$, and $\bar{CM}$ depends on the choice of parameters $\left(a_{n},b_{n}\right)$. Selecting semi-metric *d* and kernel Γ also influences efficiency. In particular, Γ is chosen to satisfy (**Co**3), while *d* controls the smoothing level of the functional predictors $I_{i}$. To examine the feasibility of the selector algorithm discussed in Section 4 we simulate with $a_{i}=C\;i^{-\upsilon }$, where υ is chosen from a 10 equidistant grid in (0,1) and $C=\max d_{i}\left(\theta ,I_{i}\right)$. Regarding the kernel Γ, we employ a quadratic kernel on (0,1), which is consistent with (**Co**3) and frequently used in nonparametric functional statistics. Moreover, the PCA metric proves especially suitable for cases in which the explanatory curves $I_{i}$ exhibit discontinuities. In this empirical analysis, we proceed with the PCA associated with the third eigenfunction. Finally we point out that we took $b_{n}=a_{n}=C\;n^{-\upsilon }$ for the estimators $\tilde{CM}$, and $\bar{CM}$, υ being selected by the same manner as in the estimator $\hat{CM}$.

To compare the effectiveness of the estimators, we compute their mean square error (MSE) across all simulated scenarios,

(12) $$\mathrm{MSE}\left(\ddot{CM}\right)=\frac{1}{n}\;\sum_{i=1}^{n}{\left(O_{i}-\ddot{CM}\left(I_{i}\right)\right)}^{2},$$

where $\ddot{CM}$ can represent either $\hat{CM}$, $\bar{CM}$, or $\tilde{CM}$. This metric enables us to contrast their performance under varying distributional assumptions dependence levels (GFAR(2) (Gaussian kernel based FAR(2)), WFAR(2) (Wiener kernel based FAR(2)), and FARCH(1)), signal-to-noise ratios, or outliers contamination. The results are reported in Table 1, Table 2 and Table 3.

The effectiveness of these estimators is substantially influenced by both the structure of the functional time series and the level of correlation. In addition, the predictor’s accuracy depends on the choice of nonparametric modeling. Nevertheless, empirical findings indicate that the recursive approach generally surpasses the NW method in terms of precision and that the $L^{1}$ method exhibits more stable mean squared error (MSE) variability compared to double-kernel techniques. Consequently, the estimator $\hat{CM}$ emerges as notably precise and robust since it combines the advantages of recursive algorithms with those of $L^{1}$-based techniques. Finally, we observe that all considered functional estimators are straightforward to implement and maintain acceptable accuracy across a variety of scenarios.

## 6. A Real Data Analysis

The purpose of this section is to evaluate how the $L^{1}$-recursive estimator of the conditional mode predictor performs in comparison with other recursive approaches. Specifically, we juxtapose it with recursive estimators of both the conditional mean and the conditional median. To conduct this forecasting problem, we rely on an environmental functional time series dataset. In particular, we focus on predicting air quality at a predetermined lead time by exploiting historical observations. The recursive predictors considered in this study are

$$\left(\mathrm{cond}.\mathrm{mean}\right)\;\hat{ME}\left(\theta \right)=\frac{\sum_{i=1}^{n}\Gamma \left(a_{i}^{-1}d\left(\theta ,I_{i}\right)\right)\;O_{i}}{\sum_{i=1}^{n}\Gamma \left(a_{i}^{-1}d\left(\theta ,I_{i}\right)\right)},$$

and

$$\left(\mathrm{cond}.\mathrm{median}\right)\;\hat{CM}\left(\theta \right)=\arg\min_{y}\frac{\sum_{i=1}^{n}\Gamma \left(a_{i}^{-1}d\left(\theta ,I_{i}\right)\right)\;\left|O_{i}-y\right|}{\sum_{j=1}^{n}\Gamma \left(a_{i}^{-1}d\left(\theta ,I_{j}\right)\right)}.$$

The dataset utilized for this comparative analysis is available online at https://gaftp.epa.gov/castnet (accessed on 8 January 2025). It contains information pertaining to the city of Bondville in Champaign, IL, USA. The monitoring station in question has the following geographical attributes, as shown in the following table.
**Country****Sate****County****Code of Station****Geographical Coordinates**USAlllinoisChampaignBVL13040.051981–88.372495

**Figure 4:**
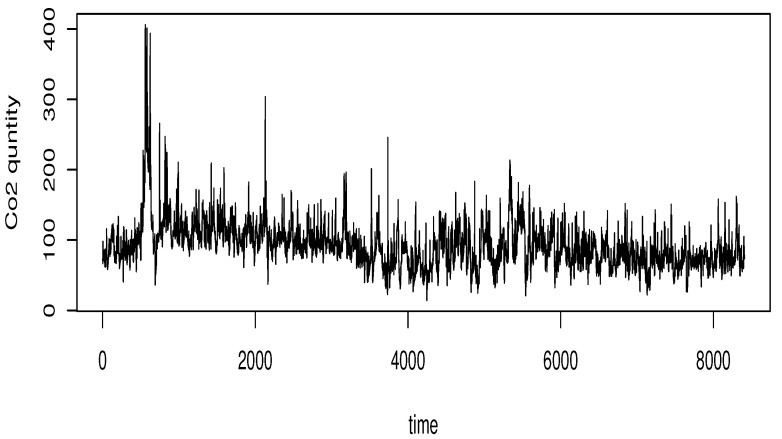
The CO_2_ emission during 2024.

The dataset was recorded at hourly intervals from January through December 2024. The original set of observations is illustrated in Figure 4.

Recall that predicting ozone levels based on CO_2_ concentrations is crucial for environmental sustainability. In Champaign, a city with a mix of urban traffic and surrounding agricultural activity, ozone levels can spike during hot, stagnant summer days, worsening air quality. Therefore, air quality in this region is significantly influenced by seasonal variations. Naturally, this effect can be managed by applying suitable seasonal data preprocessing techniques. Initially, we replaced missing values with the average of the nearest four values and employed correlation and causality analysis to pinpoint the right covariate variables. Following this preprocessing, we concluded that predicting CO_2_ emissions three hours ahead using the past 24 h of historical data is more advantageous. To do that, we segment the 8736-h dataset into N+1=364 intervals, each denoted by $I_{i}$, and each interval $I_{i}$ spans 24 h (i.e., one full day). Following this procedure, we define the output variable as

(13) $$O_{i}=I_{i+1}\left(3\right).$$

**Figure 5:**
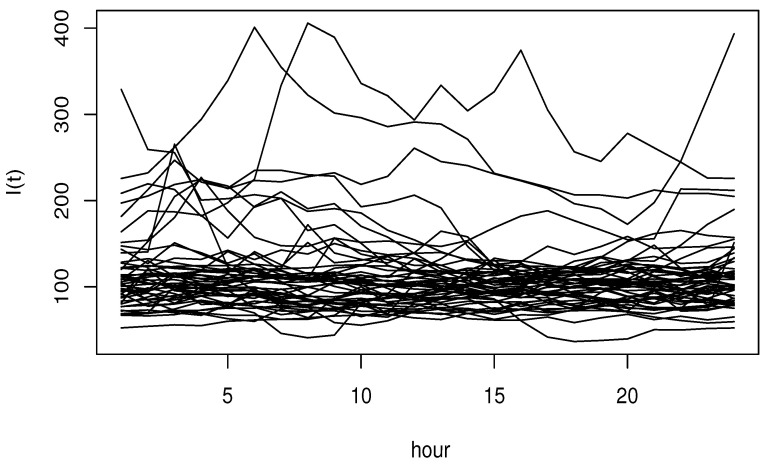
A sample of 50 curves of daily CO_2_ emission.

The curve data are given in the following graph (Figure 5).

To construct the estimators $\hat{ME}$, $\hat{CM}$, and $\hat{MO}$, we maintain the same smoothing approach, the same kernel function, and the same distance metric (PCA metric associated to the third eigenfunction). We subsequently evaluate and compare these estimators using the following procedure:

- *Step 1.* Randomly partition the dataset into two parts:
  – A training set, ${\left\{\left(I_{j},O_{j}\right)\right\}}_{j\in J}$, consisting of 300 observations;
  – A test set, ${\left\{\left(I_{i},O_{i}\right)\right\}}_{i\in I}$, consisting of 64 observations.
- *Step 2.* For each $I_{i}$ in the training set, predict the corresponding response $O_{i}$ by applying:
  – **Method 1 (Conditional mean):**
    $$\hat{O_{i}^{ME}}=\hat{ME}\left(I_{i}\right);$$
  – **Method 2 (Conditional mode):**
    $$\hat{O_{i}^{MO}}=\hat{MO}\left(I_{i}\right);$$
  – **Method 3 (Conditional median):**
    $$\hat{O_{i}^{CM}}=\hat{CM}\left(I_{i}\right).$$
- *Step 3.* For each $I_{\mathrm{new}}$ in the test set, identify
  $$i^{*}=\arg\min_{I_{i}\;\in \;\mathrm{training}\;\mathrm{set}\;J}d\left(I_{\mathrm{new}},I_{i}\right),$$
  where d(·,·) denotes the chosen distance function.
- *Step 4.* Use the identified index $i^{*}$ to predict $O_{\mathrm{new}}$:
  – **Method 1 (Conditional mean):**
    $$\hat{O_{\mathrm{new}}^{ME}}=\hat{ME}\left(I_{i^{*}}\right);$$
  – **Method 2 (Conditional mode):**
    $$\hat{O_{\mathrm{new}}^{MO}}=\hat{MO}\left(I_{i^{*}}\right);$$
  – **Method 3 (Conditional median):**
    $$\hat{O_{\mathrm{new}}^{CM}}=\hat{CM}\left(I_{i^{*}}\right).$$
- *Step 5.* To assess the prediction accuracy among the methods, compute the square root of the mean squared error (SMSE):
  $$SMSE=\sqrt{\frac{1}{64}\sum_{i\in \mathrm{Test}\;\mathrm{set}}{\left(O_{i}-\hat{T}\left(I_{i}\right)\right)}^{2}},$$
  where $\hat{T}$ can be $\hat{ME}$, $\hat{CM}$, or $\hat{MO}$.
- *Step 6.* Plot the actual response values versus the predicted values for each method.

**Figure 6:**
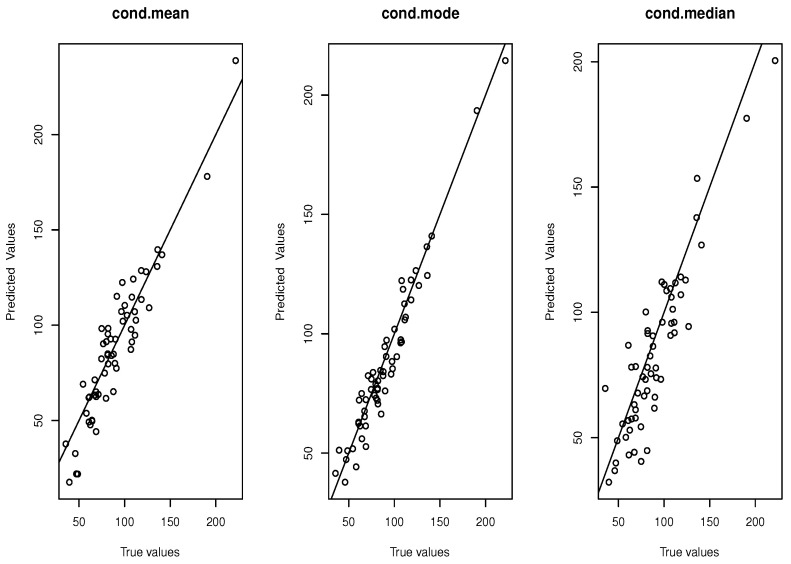
Comparison between the three predictors.

Consistent with our expectations, the $L^{1}$-based recursive predictor $\hat{CM}$ demonstrates superior performance compared to the alternative models, $\hat{ME}$ and $\hat{CM}$, see Figure 6. This improvement in predictive accuracy is notably significant. To support this assertion, we computed the standardized mean squared error (SMSE). The SMSE for $\hat{CM}$ was 3.26, while $\hat{ME}$ and the second instance of $\hat{CM}$ yielded SMSE values of 5.42 and 4.87, respectively. These predictive error measures are broadly consistent with the findings reported in [35], although one must consider the considerable differences in the climatic conditions of the regions studied. Moreover, to evaluate the sensitivity of the proposed predictors to various parameter settings, we re-ran the algorithm using the $L^{2}$ metric associated with the B-spline basis function, as well as the β-kernel with shape parameters (2,3). Subsequently, we computed the SMSE for each scenario.

Once again, $\hat{CM}$ appears to outperform the other models, $\hat{ME}$ and $\hat{CM}$. Table 4 outlines the SMSE error for the different situations. It is evident that prediction is significantly influenced by the chosen parameters of the estimators. However, the choice of the metric has a greater impact compared to the choice of the kernel. It is clear that the PCA metric is more suitable for these data. This conclusion confirms the connection between the metric and the smoothing level of the curves. In fact, using the spline metric for discontinuous curves can over-smooth the functional covariate, leading to less accurate outcomes.

## 7. Conclusions

This work introduces a new predictor based on the estimation of the $L^{1}$-modal regression, constructed by means of a recursive procedure. The theoretical discussion provides the essential mathematical underpinnings that enable the straightforward, practical application of the proposed estimator. More specifically, we establish its asymptotic behavior under the fts-ergodic assumption, an alternative condition to the conventional correlation-based criteria.

Empirical evidence from artificial and real datasets confirms that the outlined implementation accommodates the theoretical assumptions. In particular, the accuracy of the estimator depends on the degree of correlation in the data, the smoothness of the underlying nonparametric model, and the careful selection of tuning parameters such as kernel and bandwidth. Notably, combining the $L^{1}$ framework with a recursive approach offers improvements in terms of robustness and predictive precision.

In addition to these findings, the present study highlights several potential avenues for future investigation. One promising direction involves identifying the asymptotic distribution of the normalized estimator under various forms of fts, such as association or Markovian sequences. Another important extension concerns spatial modeling, which considers the geographical arrangement of the observations and supports more intricate prediction tasks. Although these extensions primarily focus on dependencies in the data, further generalizations to other smoothing methods, including the kNN approach, local linear estimators, and semi-partial linear techniques, remain equally compelling.

## 8. Proof of Propositions

**Proof of Lemma 2.**  First, we define

$$\Gamma_{i}=\Gamma \left(a_{i}^{-1}d\left(\theta ,I_{i}\right)\right)\;and\;L_{i}=\Gamma_{i}-I\;E\left[\Gamma_{i}|F_{i-1}\right].$$

Thus,

$$\begin{array}{c} {\hat{Q}}_{D}\left(\theta \right)-{\bar{Q}}_{D}\left(\theta \right)=\frac{1}{n\xi_{n}}\sum_{i=1}^{n}L_{i}. \end{array}$$

As L is a martingale difference for q≥2,

$$\begin{array}{ccc} I\;E\left[L_{i}^{q}|F_{i-1}\right] & \leq & CI\;E\left[L_{i}^{2}|F_{i-1}\right]\\ \;\\ \; & \leq & CI\;E\left[L_{i}^{2}|F_{i-1}\right]\\ \;\\ \; & < & CI\;P(I_{i}\in B\left(\theta ,a_{i}\right)|F_{i-1})\\ \;\\ \; & \leq & C\xi_{i}\left(\theta ,a_{i}\right). \end{array}$$

Now, apply the exponential to obtain

$$\begin{array}{ccc} I\;P\left\{\left|{\hat{Q}}_{D}\left(\theta \right)-{\bar{Q}}_{D}\left(\theta \right)\right|>\varepsilon \right\} & = & I\;P\left\{\left|\frac{1}{n\xi_{n}}\sum_{i=1}^{n}L_{i}\right|>\varepsilon \right\}\\ \; & \leq & 2\exp\left\{-\frac{\varepsilon^{2}n^{2}\xi_{n}^{2}\left(\theta \right)}{2(\zeta_{n}+C\varepsilon n\xi_{n})}\right\}\\ \; & = & 2\exp\left\{\frac{-\varepsilon^{2}n^{2}\xi_{n}^{2}\left(\theta \right)}{2\zeta_{n}}\left(\frac{1}{1+\frac{C\varepsilon n\xi_{n}}{\zeta_{n}}}\right)\right\}. \end{array}$$

Putting $\varepsilon =\epsilon_{0}\frac{\sqrt{\zeta_{n}\ln n}}{n\xi_{n}}$, then,

$$I\;P\left\{\left|{\hat{Q}}_{D}\left(\theta \right)-{\bar{Q}}_{D}\left(\theta \right)\right|>\epsilon_{0}\frac{\sqrt{\zeta_{n}\ln n}}{n\xi_{n}}\right\}\leq 2\exp\left\{\frac{-\epsilon_{0}^{2}\ln n}{2}\left(\frac{1}{1+\frac{C\epsilon_{0}\sqrt{\ln n}}{\sqrt{\zeta_{n}}}}\right)\right\}.$$

Since

$$\frac{\ln n}{\zeta_{n}}\leq \frac{\ln n}{Cn\xi_{n}}\leq C^{\prime }\frac{\ln n}{n\xi_{n}}\frac{\zeta_{n}}{n\xi_{n}}.$$

Therefore,

$$\lim_{n\mapsto \infty }\frac{\ln n}{\zeta_{n}}=0.$$

This implies that

$$I\;P\left\{\left|{\hat{Q}}_{D}\left(\theta \right)-{\bar{Q}}_{D}\left(\theta \right)\right|>\epsilon_{0}\frac{\sqrt{\zeta_{n}\ln n}}{n\xi_{n}}\right\}\leq 2\exp\left\{\frac{-\epsilon_{0}^{2}\ln n}{2C}\right\}\leq 2n^{-C^{\prime }\epsilon_{0}^{2}}.$$

Hence,

$${\hat{Q}}_{D}\left(\theta \right)-{\bar{Q}}_{D}\left(\theta \right)=O\left(\sqrt{\frac{\zeta_{n}\ln n}{n^{2}\xi_{n}^{2}}}\right),\;a.co.$$

For the second result, we have the following for C>0

$$\;0<C\frac{\sum_{i=1}^{n}I\;P\left(I_{i}\in B\left(\theta ,a_{i}\right)|F_{i-1}\right)}{\sum_{i=1}^{n}I\;P\left(I_{i}\in B\left(\theta ,a_{i}\right)\right)}\leq {\bar{Q}}_{D}\left(\theta \right)\leq \left|{\bar{Q}}_{D}\left(\theta \right)-{\hat{Q}}_{D}\left(\theta \right)\right|+{\hat{Q}}_{D}\left(\theta \right).$$

Thus,

$$C\frac{\sum_{i=1}^{n}I\;P\left(I_{i}\in B\left(\theta ,a_{i}\right)|F_{i-1}\right)}{\sum_{i=1}^{n}I\;P\left(I_{i}\in B\left(\theta ,a_{i}\right)\right)}-\left|{\hat{Q}}_{D}\left(\theta \right)-{\bar{Q}}_{D}\left(\theta \right)\right|<{\hat{Q}}_{D}\left(\theta \right).$$

So,

$$\begin{array}{c} I\;P\left({\hat{Q}}_{D}\left(\theta \right)\leq \frac{C}{2}\right)\\ \;\leq \;I\;P\left(C\frac{\sum_{i=1}^{n}I\;P\left(I_{i}\in B\left(\theta ,a_{i}\right)|F_{i-1}\right)}{\sum_{i=1}^{n}I\;P\left(I_{i}\in B\left(\theta ,a_{i}\right)\right)}-\left|{\hat{Q}}_{D}\left(\theta \right)-{\bar{Q}}_{D}\left(\theta \right)\right|<\frac{C}{2}\right)\\ \;\leq \;I\;P\left(\left|C\frac{\sum_{i=1}^{n}I\;P\left(I_{i}\in B\left(\theta ,a_{i}\right)|F_{i-1}\right)}{\sum_{i=1}^{n}I\;P\left(I_{i}\in B\left(\theta ,a_{i}\right)\right)}-\left|{\hat{Q}}_{D}\left(\theta \right)-{\bar{Q}}_{D}\left(\theta \right)\right|-C\right|>\frac{C}{2}\right). \end{array}$$

It follows that

$$\begin{array}{c} \sum_{n}I\;P\left({\hat{Q}}_{D}\left(\theta \right)\leq \frac{C}{2}\right)\\ \;\leq \;\sum_{n}I\;P\left(\left|C\frac{\sum_{i=1}^{n}I\;P\left(I_{i}\in B\left(\theta ,a_{i}\right)|F_{i-1}\right)}{\sum_{i=1}^{n}I\;P\left(I_{i}\in B\left(\theta ,a_{i}\right)\right)}-\left|{\hat{Q}}_{D}\left(\theta \right)-{\bar{Q}}_{D}\theta )\right|-C\right|>\frac{C}{2}\right)<\infty . \end{array}$$

Finally, from the first result, we obtain

$$\sum_{n}I\;P\left({\bar{Q}}_{D}\left(\theta \right)\leq \frac{C}{2}\right)<\infty .$$

□

**Proof of Propositions 1.**  Let

$$\gamma_{n}={\hat{Qu}}_{p}\left(\theta \right)-Qu_{p}\left(\theta \right).$$

Clearly, $B_{n}\left(\gamma_{n}\right)=0$. Now, we check

(14) $$A_{n}=o_{a.co.}\left(1\right),$$

and there exist M,λ>0 such that

(15) $$\underset{|\gamma |\leq M}{\sup}\left|B_{n}\left(\gamma \right)+\lambda \gamma -A_{n}\right|=o_{a.co.}\left(1\right).$$

**For (14)**, we evaluate

$$A_{n}-\bar{A_{n}}=O_{a.co.}\left(\sqrt{\frac{\zeta_{n}\ln n}{n^{2}\xi_{n}^{2}}}\right)\;\mathrm{and}\;\bar{A_{n}}=O\left(\frac{1}{\zeta_{n}}\sum_{i=1}^{n}a_{i}^{b}\xi_{i}\left(\theta ,a_{i}\right)\right),$$

where

$$\bar{A_{n}}=\frac{1}{n\xi_{n}}\sum_{i=1}^{n}I\;E\left[\left(p-1\;I_{\left[O_{i}\leq Qu_{p}\left(\theta \right)\right]}\right)\Gamma_{i}|F_{i-1}\right].$$

Firstly, we have

$$\begin{array}{ccc} \forall \epsilon >0\;I\;P\left\{\left|A_{n}-\bar{A_{n}}\right|>\varepsilon \right\} & = & I\;P\left\{\left|\frac{1}{n\xi_{n}}\sum_{i=1}^{n}\Psi_{i}\right|>\varepsilon \right\}\\ \; & \leq & I\;P\left\{\left|\sum_{i=1}^{n}\Psi_{i}\right|>\varepsilon n\xi_{n}\right\}, \end{array}$$

with

$$\Psi_{i}=\frac{1}{n\xi_{n}}\left(p-1\;I_{\left[O_{i}\leq Qu_{p}\left(\theta \right)\right]}\right)\Gamma_{i}-I\;E\left[\left(p-1\;I_{\left[O_{i}\leq Qu_{p}\left(\theta \right)\right]}\right)\Gamma_{i}|F_{i-1}\right].$$

Clearly, $\Psi_{i}$ is a martingale difference with respect to the σ-algebra, and ${\left(F_{i-1}\right)}_{i}$ satisfies the following for q≥2

$$\begin{array}{ccc} I\;E\left[\Psi_{i}^{q}|F_{i-1}\right] & \leq & CI\;E\left[\Psi_{i}^{2}|F_{i-1}\right]\\ \;\\ \; & \leq & CI\;E\left[\Gamma_{i}^{2}|F_{i-1}\right]\\ \;\\ \; & < & CI\;P(I_{i}\in B\left(\theta ,a_{i}\right)|F_{i-1})\\ \;\\ \; & \leq & C\xi_{i}\left(\theta ,a_{i}\right). \end{array}$$

Thus,

$$\begin{array}{ccc} I\;P\left\{\left|A_{n}-\bar{A_{n}}\right|>\varepsilon \right\} & = & I\;P\left\{\left|\frac{1}{n\xi_{n}}\sum_{i=1}^{n}\Psi_{i}\right|>\varepsilon \right\}\\ \; & \leq & 2\exp\left\{-\frac{\varepsilon^{2}n^{2}\xi_{n}^{2}}{2(\zeta_{n}+C\varepsilon n\xi_{n})}\right\}\\ \; & = & 2\exp\left\{\frac{-\varepsilon^{2}n^{2}\xi_{n}^{2}}{2\zeta_{n}}\left(\frac{1}{1+\frac{C\varepsilon n\xi_{n}}{\zeta_{n}}}\right)\right\}. \end{array}$$

So, for $\varepsilon =\epsilon_{0}\frac{\sqrt{\zeta_{n}\ln n}}{n\xi_{n}}$, we have,

$$I\;P\left\{\left|A_{n}-\bar{A_{n}}\right|>\epsilon_{0}\frac{\sqrt{\zeta_{n}\ln n}}{n\xi_{n}}\right\}\leq 2\exp\left\{\frac{-\epsilon_{0}^{2}\ln n}{2}\left(\frac{1}{1+\frac{C\epsilon_{0}\sqrt{\ln n}}{\sqrt{\zeta_{n}}}}\right)\right\}.$$

Since

$$\frac{\ln n}{\zeta_{n}}\leq \frac{\ln n}{Cn\xi_{n}}\leq C^{\prime }\frac{\ln n}{n\xi_{n}}\frac{\zeta_{n}}{n\xi_{n}}.$$

Then,

$$\lim_{n\mapsto \infty }\frac{\ln n}{\zeta_{n}}=0.$$

Therefore,

$$I\;P\left\{\left|A_{n}-\bar{A_{n}}\right|>\epsilon_{0}\frac{\sqrt{\zeta_{n}\ln n}}{n\xi_{n}}\right\}\leq 2\exp\left\{\frac{-\epsilon_{0}^{2}\ln n}{2C}\right\}\leq 2n^{-C^{\prime }\epsilon_{0}^{2}}.$$

For the second one, we have

$${1\;I}_{B(\theta ,)}(I_{1})|G(t,I_{i})-G(t,\theta )|\leq Ca_{i}^{b}$$

Then,

(16) $$\begin{array}{ccc} \bar{A_{n}} & = & \frac{1}{n\xi_{n}}\sum_{i=1}^{n}I\;E\left[\left(p-1\;I_{\left[O_{i}\leq Qu_{p}\left(\theta \right)\right]}\right)\Gamma_{i}|F_{i-1}\right]\\ \; & = & \frac{1}{n\xi_{n}}\sum_{i=1}^{n}\left(I\;E\left[\Gamma_{i}\left(G\left(Qu_{p}\left(\theta \right)|\theta \right)-I\;E\left[1\;I_{\left[O_{i}\leq Qu_{p}\left(\theta \right)\right]}|I_{i}\right]\right)|F_{i-1}\right]\right)\\ \; & \leq & \frac{1}{n\xi_{n}}\sum_{i=1}^{n}I\;E\left[\Gamma_{i}{1\;I}_{B(\theta ,a_{i})}\left(I_{i}\right)\left|G\left(Qu_{p}\left(\theta \right)|\theta \right)-G\left(Qu_{p}\left(\theta \right)|I_{i}\right)\right||F_{i-1}\right].\\ \; & \leq & \frac{1}{n\xi_{n}}\sum_{i=1}^{n}a_{i}^{b}I\;E\left[\Gamma_{i}|F_{i-1}\right]. \end{array}$$

Consequently,

$$\left|A_{n}-\bar{A_{n}}\right|=O_{a.co.}\left(\sqrt{\frac{\zeta_{n}\ln n}{n^{2}\xi_{n}^{2}}}\right)$$

and

$$\bar{A_{n}}=O\left(\frac{1}{\zeta_{n}}\sum_{i=1}^{n}a_{i}^{b}\xi_{i}\left(\theta ,a_{i}\right)\right).$$

**For (15)**, similar to (14), we split the required result into parts

(17) $$\underset{|\gamma |\leq M}{\sup}\left|B_{n}\left(\gamma \right)-A_{n}-\frac{1}{n\xi_{n}}\sum_{i=1}^{n}I\;E\left[\left(B_{n}\left(\gamma \right)-A_{n}\right)|F_{i-1}\right]\right|=O_{a.co.}\left(\sqrt{\frac{\zeta_{n}\ln n}{n^{2}\xi_{n}^{2}}}\right),$$

and the bias term

(18) $$\underset{|\gamma |\leq M}{\sup}\left|\frac{1}{n\xi_{n}}\sum_{i=1}^{n}I\;E\left[B_{n}\left(\gamma \right)-A_{n}|F_{i-1}\right]+g\left(Qu_{p}\left(\theta \right)\right)\gamma \right|=O\left(\frac{1}{\zeta_{n}}\sum_{i=1}^{n}a_{i}^{b}\xi_{i}\left(\theta ,a_{i}\right)\right).$$

Let us start with the dispersion term in (17). We employ the compactness of the interval [−M,M] and write

$$\left[-M,M\right]\subset \overset{d_{n}}{\underset{j=1}{⋃}}\left[\gamma_{j}-l_{n},\gamma_{j}+l_{n}\right],\;\mathrm{for}\;\;\gamma_{j}\in \left[-M,M\right]\mathrm{and}\;l_{n}=d_{n}^{-1}=1/\sqrt{n}.$$

So, for all γ∈[−M,M], we put $j\left(\gamma \right)=\arg\min_{j}\left|\gamma -\gamma_{j}\right|$ and use the monotony of $B_{n}\left(\cdot \right)$ and $I\;E\left[B_{n}\left(\gamma \right)|F_{i-1}\right]$, which leads to the following for all $1\leq j\leq d_{n}$,

$$B_{n}\left(\gamma_{j}+l_{n}\right)\leq \underset{y\in (\gamma_{j}-l_{n},\gamma_{j}+l_{n})}{\sup}B_{n}\left(\gamma \right)\leq B_{n}\left(\gamma_{j}-l_{n}\right)$$

and

$$I\;E\left[B_{n}\left(\gamma_{j}+l_{n}\right)|F_{i-1}\right]\leq \underset{\gamma \in (\gamma_{j}+l_{n},\gamma_{j}+l_{n})}{\sup}I\;E\left[B_{n}\left(\gamma \right)|F_{i-1}\right]\leq I\;E\left[B_{n}\left(\gamma_{j}-l_{n}\right)|F_{i-1}\right].$$

We deduce, for any $\gamma_{1},\gamma_{2}\in \left[-M,M\right]$,

$$\left|\frac{1}{n\xi_{n}}\sum_{i=1}^{n}I\;E\left[B_{n}\left(\gamma_{1}\right)|F_{i-1}\right]-\frac{1}{n\xi_{n}}\sum_{i=1}^{n}I\;E\left[B_{n}\left(\gamma_{2}\right)|F_{i-1}\right]\right|\leq C{\left|\gamma_{1}-\gamma_{2}\right|}^{b}{\bar{Q}}_{D}\left(x\right).$$

It follows that

$$\begin{array}{c} \underset{|\gamma |\leq M}{\sup}\left|B_{n}\left(\gamma \right)-A_{n}-\frac{1}{n\xi_{n}}\sum_{i=1}^{n}I\;E\left[\left(B_{n}\left(\gamma \right)-A_{n}\right)|F_{i-1}\right]\right|\\ \;\leq \;\max_{1\leq j\leq d_{n}}\max_{z\in \{\gamma_{j}-l_{n},\gamma_{j}+l_{n}\}}\left|B_{n}\left(z\right)-A_{n}-\frac{1}{n\xi_{n}}\sum_{i=1}^{n}I\;E\left[\left(B_{n}\left(z\right)-A_{n}\right)|F_{i-1}\right]\right|\\ \;+2^{b}Cl_{n}^{b}{\bar{Q}}_{D}\left(x\right). \end{array}$$

Concerning $l_{n}^{b}$, we write

$$\begin{array}{ccc} \frac{l_{n}^{b}}{\sqrt{\frac{\zeta_{n}\log n}{n^{2}\xi_{n}^{2}}}} & = & \frac{l_{n}^{b}n\xi_{n}}{\sqrt{\zeta_{n}\log n}}\\ \;\\ \; & = & \frac{n\xi_{n}}{\sqrt{n\zeta_{n}\log n}}\\ \;\\ \; & = & \sqrt{\frac{\sum_{i=1}^{n}\xi \left(\theta ,a_{i}\right)}{n}}\sqrt{\frac{\sum_{i=1}^{n}\xi \left(\theta ,a_{i}\right)}{\sum_{i=1}^{n}\xi_{i}\left(\theta ,a_{i}\right)}}\sqrt{\frac{1}{\log n}}. \end{array}$$

Furthermore, as $\xi (\theta ,a_{i})\leq 1$ we have, for all *n*,

$$\frac{\sum_{i=1}^{n}\xi \left(\theta ,a_{i}\right)}{n}\leq 1,$$

and by (**(C1))(iii)**

$$\lim_{n\mapsto \infty }\frac{\sum_{i=1}^{n}\xi \left(\theta ,a_{i}\right)}{\sum_{i=1}^{n}\xi_{i}\left(\theta ,a_{i}\right)}\leq \lim_{n\mapsto \infty }\frac{\sum_{i=1}^{n}\xi \left(\theta ,a_{i}\right)}{\sum_{i=1}^{n}I\;P\left(I_{i}\in B\left(x,r_{i}\right)|F_{i-1}\right)}=1.$$

Finally, we can write

$$l_{n}^{b}=O\left(\sqrt{\frac{\zeta_{n}\log n}{n^{2}\xi_{n}^{2}}}\right).$$

Dealing with

$$\underset{|\gamma |\leq M}{\sup}\left|B_{n}\left(\gamma_{j}\right)-A_{n}-\frac{1}{n\xi_{n}}\sum_{i=1}^{n}I\;E\left[B_{n}\left(\gamma_{j}\right)-A_{n}|F_{i-1}\right]\right|,$$

we set

$$B_{n}\left(\gamma_{j}\right)-A_{n}-\frac{1}{n\xi_{n}}\sum_{i=1}^{n}I\;E\left[B_{n}\left(\gamma_{j}\right)-A_{n}|\right]=\frac{1}{n\xi_{n}}\sum_{i=1}^{n}\Upsilon_{i},$$

with

$$\begin{array}{ccc} \Upsilon_{i} & = & \left(1\;I_{O_{i}\leq Qu_{p}\left(\theta \right)}-1\;I_{O_{i}\leq \gamma_{j}+Qu_{p}\left(\theta \right)}\right)\Gamma_{i}\\ \; & \; & -I\;E\left[\left(1\;I_{O_{i}\leq Qu_{p}\left(\theta \right)}-1\;I_{O_{i}\leq \gamma_{j}+Qu_{p}\left(\theta \right)}\right)\Gamma_{i}|F_{i-1}\right]. \end{array}$$

As in $A_{n}$,

$$\begin{array}{ccc} I\;E\left[\Upsilon_{i}^{q}|F_{i-1}\right] & \leq & CI\;E\left[\Upsilon_{i}^{2}|F_{i-1}\right]\\ \;\\ \; & \leq & CI\;E\left[\Gamma_{i}^{2}|F_{i-1}\right]\\ \;\\ \; & < & CI\;P(I_{i}\in B\left(\theta ,a_{i}\right)|F_{i-1})\\ \;\\ \; & \leq & C\xi_{i}\left(\theta ,a_{i}\right). \end{array}$$

Therefore,

$$\begin{array}{c} I\;P\left\{\left|B_{n}\left(\gamma_{j}\right)-A_{n}-\frac{1}{n\xi_{n}}\sum_{i=1}^{n}I\;E\left[B_{n}\left(\gamma_{j}\right)-A_{n}|F_{i-1}\right]\right|>\epsilon_{0}\frac{\sqrt{\zeta_{n}\ln n}}{n\xi_{n}}\right\}\\ \leq 2\exp\left\{\frac{-\epsilon_{0}^{2}\ln n}{2C}\right\}\leq 2n^{-C^{\prime }\epsilon_{0}^{2}}. \end{array}$$

Consequently,

$$\begin{array}{c} \sum_{n}I\;P\left(\underset{|\gamma |\leq M}{\sup}\left|B_{n}\left(\gamma_{j(\gamma )}\right)-A_{n}-\frac{1}{n\xi_{n}}\sum_{i=1}^{n}I\;E\left[B_{n}\left(\gamma_{j(\gamma )}\right)-A_{n}\right]\right|\geq \epsilon_{0}\left(\frac{\sqrt{\zeta_{n}\ln n}}{n\xi_{n}}\right)\right)\\ \;\leq \;\sum_{n}d_{n}\max_{j}I\;P\left(\left|B_{n}\left(\gamma_{j}\right)-A_{n}-\frac{1}{n\xi_{n}}\sum_{i=1}^{n}I\;E\left[B_{n}\left(\gamma_{j}\right)-A_{n}\right]\right|\geq \epsilon_{0}\left(\frac{\sqrt{\zeta_{n}\ln n}}{n\xi_{n}}\right)\right)<\infty , \end{array}$$

implying (17). Concerning (18), we write

$$\begin{array}{c} \frac{1}{n\xi_{n}}\sum_{i=1}^{n}I\;E\left[B_{n}\left(\gamma \right)-A_{n}|F_{i-1}\right]\\ \;=\;-\frac{1}{n\xi_{n}}\sum_{i=1}^{n}I\;E\left[\left(1\;I_{\left[O_{1}\leq \gamma +Qu_{p}\left(\theta \right)\right]}-1\;I_{\left[O_{1}\leq Qu_{p}\left(\theta \right)\right]}\right)\Gamma_{i}|F_{i-1}\right]\\ \;=\;-\frac{1}{n\xi_{n}}\sum_{i=1}^{n}I\;E\left[\left(G\left(\gamma +Qu_{p}\left(\theta \right)|I_{1}\right)-G\left(Qu_{p}\left(\theta \right)|I_{1}\right)\right)\Gamma_{i}|F_{i-1}\right]\\ \;=\;-\frac{1}{n\xi_{n}}\sum_{i=1}^{n}I\;E\left[\left(G\left(\gamma +Qu_{p}\left(\theta \right)|\theta \right)-g\left(Qu_{p}\left(\theta \right)|\theta \right)\right)\Gamma_{i}|F_{i-1}\right]+O\left(a_{i}^{b}\right)\\ \;=\;-\frac{\gamma g(Qu_{p}\left(\theta \right)|\theta )}{\xi_{n}}\frac{1}{n}\sum_{i=1}^{n}I\;E\left[\Gamma_{i}|F_{i-1}\right]+O\left(\frac{1}{\zeta_{n}}\sum_{i=1}^{n}a_{i}^{b}\xi_{i}\left(\theta ,a_{i}\right)\right)+o\left(\gamma \right). \end{array}$$

It follows that

$$\begin{array}{c} I\;E\left[B_{n}\left(\gamma \right)-A_{n}|F_{i-1}\right]\\ \;=\;-g\left(Qu_{p}|\theta \right){\bar{Q}}_{D}\left(x\right)\gamma +O\left(\frac{1}{\zeta_{n}}\sum_{i=1}^{n}a_{i}^{b}\xi_{i}\left(\theta ,a_{i}\right)\right)+o\left(\gamma \right). \end{array}$$

Therefore, the Bahadur representation of ${\hat{Qu}}_{p}\left(\theta \right)-Qu_{p}\left(\theta \right)$ is

$${\hat{Qu}}_{p}\left(\theta \right)-Qu_{p}\left(\theta \right)=\frac{1}{g(Qu_{p}|\theta )}A_{n}+O\left(\underset{|\gamma |\leq M}{\sup}\left|B_{n}\left(\gamma \right)+\lambda \gamma -A_{n}\right|\right).$$

□

**Proof of Propositions 2.**  The uniform consistency of ${\hat{Qu}}_{p}\left(\theta \right)-Qu_{p}\left(\theta \right)$ is based on

(19) $$\underset{p\in [0,1]}{\sup}\left|A_{n}-I\;E\left[A_{n}\right]\right|=O_{a.co.}\left(\frac{\sqrt{\zeta_{n}\ln n}}{n\xi_{n}}\right),$$

and

(20) $$\underset{p\in [0,1]}{\sup}\underset{|\gamma |\leq M}{\sup}\left|B_{n}\left(\gamma \right)+g\left(Qu_{p}|\theta \right)\gamma -A_{n}\right|=O_{a.co.}\left(\frac{\sqrt{\zeta_{n}\ln n}}{n\xi_{n}}\right).$$

Since the inequalities in the bias terms are uniform on p∈[0,1]. We only focus on the dispersion terms of

$$\underset{p\in [0,1]}{\sup}\left|A_{n}\left(p\right)\right|\;\mathrm{and}\;\underset{p\in [0,1]}{\sup}\underset{|\gamma |\leq M}{\sup}\left|F_{n}\left(\gamma ,p\right)\right|,$$

where

$$A_{n}\left(p\right)=\left|A_{n}-\frac{1}{n\xi_{n}}\sum_{i=1}^{n}I\;E\left[A_{n}|F_{i-1}\right]\right|,$$

and

$$F_{n}\left(\gamma ,p\right)=\left|B_{n}\left(\gamma \right)-A_{n}-\frac{1}{n\xi_{n}}\sum_{i=1}^{n}I\;E\left[\left(B_{n}\left(\gamma \right)-A_{n}\right)|F_{i-1}\right]\right|.$$

We focus on the first-term, the second one is similar. Indeed,

$$\left[0,1\right]\subset \overset{d_{n}}{\underset{k=1}{⋃}}\left[p_{k}-l_{n},p_{k}+l_{n}\right],\;for\;\;p_{k}\;\in \left[0,1\right].$$

Next, for all p∈[0,1], we put $\eta_{p}=\arg\min_{k}\left|p-p_{k}\right|$. Then,

$$\underset{p\in [0,1]}{\sup}\left|A_{n}\left(p\right)\right|\leq \max_{1\leq j\leq d_{n}}\max_{z_{p}\in \left\{p_{j}-l_{n},p_{j}+l_{n}\right\}}\left|A_{n}\left(z_{p}\right)\right|+2^{b}Cl_{n}^{b}{\bar{Q}}_{D}\left(x\right).$$

It is shown in Lemma that for all p∈(0,1) that

$$I\;P\left(\left|A_{n}\left(z_{p}\right)\right|\geq \epsilon_{0}\left(\frac{\sqrt{\zeta_{n}\ln n}}{n\xi_{n}}\right)\right)\leq 2n^{-C^{\prime }\epsilon_{0}^{2}}.$$

Therefore,

$$\begin{array}{c} \sum_{n}I\;P\left(\underset{p\in [0,1]}{\sup}\left|A_{n}\left(p\right)\right|\geq \epsilon_{0}\left(\frac{\sqrt{\zeta_{n}\ln n}}{n\xi_{n}}\right)\right)\\ \;\leq \;\sum_{n}d_{n}\max_{j}I\;P\left(\left|A_{n}\left(p\right)\right|\geq \epsilon_{0}\left(\frac{\sqrt{\zeta_{n}\ln n}}{n\xi_{n}}\right)\right)<\infty . \end{array}$$

The uniform consistency of ($\sup_{p\in [0,1]}\left|I\;E\left[A_{n}\right]\right|$) is obtained by taking the uniform version of (16), which allows us to conclude that

$$\underset{p\in [0,1]}{\sup}\left|{\hat{Qu}}_{p}\left(\theta \right)-Qu_{p}\left(\theta \right)\right|=O\left(\frac{1}{\zeta_{n}}\sum_{i=1}^{n}a_{i}^{b}\xi_{i}\left(\theta ,a_{i}\right)\right)+O\left(\sqrt{\frac{\zeta_{n}\ln n}{n^{2}\xi_{n}^{2}}}\right).$$

□

## Figures and Tables

**Table 1:** MSE errors of the estimator $\hat{CM}$.

MF	FTS	Dist.	Het.	Hom.	SNRHet (5%)	SNRHet (40%)
MF = 1	GFAR(2)	Laplace	0.176	0.154	1.174	2.197
		Log normal	0.183	0.166	1.187	2.198
		Weibull	0.196	0.172	1.189	2.208
	WFAR(2)	Laplace	0.165	0.143	0.158	2.171
		Log normal	0.173	0.141	1.153	2.185
		Weibull	0.170	0.153	1.170	2.192
	FARCH(1)	Laplace	0.201	0.182	1.204	2.209
		Log normal	0.223	0.209	1.311	2.534
		Weibull	0.240	0.223	1.412	2.626
MF = 10	GFAR(2)	Laplace	0.256	0.261	1.353	2.413
		Log normal	0.317	0.312	1.487	2.507
		Weibull	0.296	0.542	1.618	2.698
	WFAR(2)	Laplace	0.356	0.334	1.385	0.497
		Log normal	0.432	0.513	1.635	2.758
		Weibull	0.408	0.443	1.489	2.595
	FARCH(1)	Laplace	0.513	0.523	1.641	2.691
		Log normal	0.434	0.419	1.453	2.554
		Weibull	0.504	0.514	1.562	1.516

**Table 2:** MSE errors of the estimator $\tilde{CM}$.

MF	FTS	Dist.	HeT.	Hom.	SNRHet (5%)	SNRHet (40%)
MF = 1	GFAR(2)	Laplace	1.161	1.109	2.008	4.378
		Log normal	1.304	0.963	2.789	4.678
		Weibull	3.239	2.107	3.896	4.894
	WFAR(2)	Laplace	0.876	0.403	1.097	1.856
		Log normal	0.606	0.236	1.765	2.785
		Weibull	1.690	1.327	2.045	2.976
	FARCH(1)	Laplace	2.332	1.763	2.435	4.554
		Log normal	2.204	1.398	3.971	5.861
		Weibull	5.109	3.712	5.023	6.432
MF = 10	GFAR(2)	Laplace	4.201	4.216	7.312	8.417
		Log normal	4.230	4.736	6.789	7.678
		Weibull	5.117	6.107	7.186	8.243
	WFAR(2)	Laplace	3.654	3.212	4.178	5.164
		Log normal	3.902	4.561	5.605	6.194
		Weibull	4.310	4.127	6.205	6.817
	FARCH(1)	Laplace	6.231	7.862	8.333	9.352
		Log normal	4.315	5.493	6.771	8.662
		Weibull	7.101	7.513	8.224	9.533

**Table 3:** MSE errors of the estimators $\bar{CM}$.

MF	FTS	Dist.	Het.	Hom.	SNRHet (5%)	SNRHet (40%)
MF = 1	GFAR(2)	Laplace	1.535	1.331	2.103	4.414
		Log normal	1.202	1.106	2.452	4.786
		Weibull	2.119	1.811	3.02	4.949
	WFAR(2)	Laplace	0.167	0.156	1.861	3.843
		Log normal	0.134	0.136	1.451	3.073
		Weibull	0.109	0.117	0.698	1.785
	FARCH(1)	Laplace	3.101	2.512	3.972	4.952
		Log normal	2.603	2.363	3.861	5.045
		Weibull	4.009	3.227	4.961	5.895
MF = 10	GFAR(2)	Laplace	2.552	2.312	4.132	6.447
		Log normal	2.221	3.166	5.421	8.761
		Weibull	4.191	5.823	6.211	8.991
	WFAR(2)	Laplace	2.171	2.164	4.812	6.832
		Log normal	2.142	0.162	1.412	3.264
		Weibull	2.192	2.173	3.682	5.751
	FARCH(1)	Laplace	8.112	8.521	9.128	9.921
		Log normal	4.611	4.169	6.161	10.012
		Weibull	9.018	10.271	11.031	12.185

**Table 4:** SMSE errors of the three predictors with respect to different metrics and different kernels.

Model	Metric	Kernel	SMSE
$\hat{CM}$	PCA (3th eigenfunction)	Quadratic kernel	3.26
	PCA (3th eigenfunction)	β-kernel	3.37
	8th eigenfunction	Quadratic kernel	4.03
	8th eigenfunction	β-kernel	4.11
	$L_{2}$ Spline metric	Quadratic kernel	4.39
	$L_{2}$ Spline metric	β-kernel	4.52
$\hat{ME}$	PCA (3th eigenfunction)	Quadratic kernel	5.42
	PCA (3th eigenfunction)	β-kernel	5.61
	8th eigenfunction	Quadratic kernel	7.56
	8th eigenfunction	β-kernel	8.22
	$L_{2}$ Spline metric	Quadratic kernel	6.45
	$L_{2}$ Spline metric	β-kernel	6.82
$\hat{CM}$	PCA (3th eigenfunction)	Quadratic kernel	4.87
	PCA (3th eigenfunction)	β-kernel	5.11
	8th eigenfunction	Quadratic kernel	8.62
	8th eigenfunction	β-kernel	8.34
	$L_{2}$ Spline metric	Quadratic kernel	6.12
	$L_{2}$ Spline metric	β-kernel	6.73

## Data Availability

The data used in this study are available through the link https://kilthub.cmu.edu (accessed on 2 February 2025).
